# Heart failure and cognitive impairment: a narrative review

**DOI:** 10.1093/eschf/xvag192

**Published:** 2026-07-13

**Authors:** Izabella Uchmanowicz, Heba Aldossary, Maria Próba, Erik Fung, Kenneth Faulkner, Loreena Hill, Marta Kaluzna-Oleksy, Wiktoria Szpek, Julia Maj, Asriel Juvenal Chamos, Quin Denfeld, Patryk Rewaj, Maggie Simpson, Cristiana Vitale

**Affiliations:** Department of Nursing, Faculty of Nursing and Midwifery, Wroclaw Medical University, Wroclaw, Poland; Centre for Cardiovascular Health, Edinburgh Napier University, Sighthill Campus, Edinburgh, UK; Department of Nursing, Prince Sultan Military College of Health Sciences, P.O. Box 946, Dhahran 31932, Saudi Arabia; Frances Payne Bolton School of Nursing, Case Western Reserve University, 10900 Euclid Avenue, Cleveland, OH 44106, USA; Department of Nursing, Faculty of Nursing and Midwifery, Wroclaw Medical University, Wroclaw, Poland; School of Medicine, The Hong Kong University of Science and Technology, Clear Water Bay, Hong Kong SAR, China; School of Public Health, Faculty of Medicine, Imperial College London, London, UK; Stony Brook University School of Nursing, Stony Brook, NY, USA; School of Nursing and Paramedic Science, Ulster University, Londonderry, UK; Department of Cardiology, University of Medical Sciences, Poznan, Poland; University of Medical Sciences, Poznan, Poland; Department of Nursing, Faculty of Nursing and Midwifery, Wroclaw Medical University, Wroclaw, Poland; School of Nursing and Paramedic Science, Ulster University, Londonderry, UK; School of Nursing, Oregon Health & Science University, Portland, OR, USA; Department of Nursing, Faculty of Nursing and Midwifery, Wroclaw Medical University, Wroclaw, Poland; Edinburgh Medical School, University of Edinburgh, Edinburgh, UK; Department of Human Sciences and Promotion of Quality of Life, San Raffaele Open University of Rome, Rome, Italy; Department of Cardiac and Respiratory Sciences, IRCCS San Raffaele Roma, Rome, Italy

**Keywords:** Heart failure, Cognitive impairment, Mild cognitive impairment, Dementia, Self-care, Medication adherence

## Abstract

Heart failure (HF) is a major global health burden affecting more than 64 million individuals worldwide, and is associated with substantial morbidity, mortality, and healthcare expenditure. Cognitive impairment (CI) is a prevalent and clinically consequential comorbidity, affecting approximately 40%–50% of patients with HF across both reduced and preserved ejection fraction phenotypes. Despite its high prevalence and adverse prognostic implications, CI remains systematically underdiagnosed and insufficiently addressed in routine clinical practice. The pathophysiological mechanisms linking HF and CI are multifactorial and incompletely understood, encompassing cerebral hypoperfusion secondary to reduced cardiac output, systemic neuroinflammation driven by pro-inflammatory cytokines, oxidative stress, neurohumoral dysregulation, and cerebrovascular injury. Shared cardiovascular risk factors, including atrial fibrillation, hypertension, diabetes mellitus, and obesity, further compound the risk of cognitive decline. CI exerts a profound impact on clinical outcomes in HF, independently predicting increased all-cause mortality, higher rates of hospitalization and 30-day readmission, impaired self-care capacity, and reduced medication adherence. Early and systematic identification is a reasonable clinical priority that may support optimized clinical management and informed shared decision-making. This review synthesizes current evidence on the epidemiology, pathophysiology, clinical assessment, prognostic significance, and therapeutic implications of CI in HF, and highlights key controversies and priorities for future research.

## Graphical Abstract

**Graphical Abstract:**
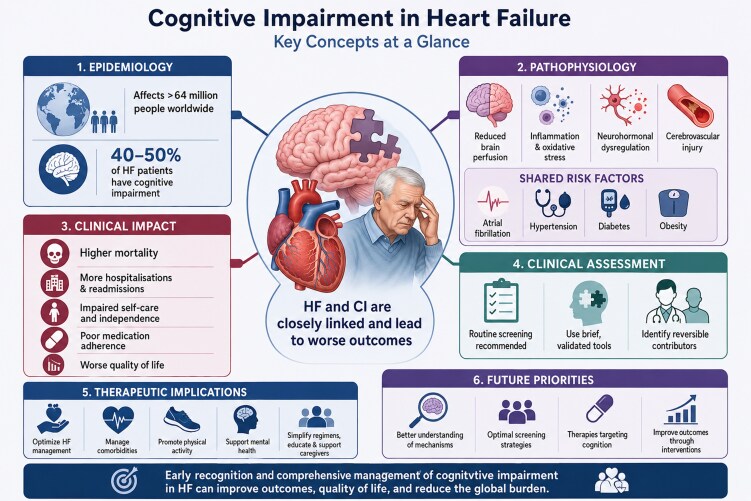

## Introduction

Heart failure (HF) is a clinical syndrome characterized by current or prior symptoms resulting from a structural or functional cardiac abnormality.^1^ Heart failure is highly prevalent, affecting more than 64 million individuals worldwide.^2,3^ Clinical management requires complex, multi-drug pharmacological regimens targeting neurohormonal activation, fluid retention, and rate or rhythm control, placing significant and sustained demands on patients’ cognitive and self-management capacities.

Cognitive impairment (CI), defined as objective evidence of deficits in one or more cognitive domains exceeding that attributable to normal ageing,^4^ and HF frequently coexist, particularly in elderly patients. Although reported prevalence varies widely according to assessment methodology, meta-analyses estimate that approximately 40% of patients with HF experience some degree of CI.^4^ Cognitive function exists on a continuum spanning from fully preserved cognition through mild cognitive impairment (MCI) to frank dementia.

Patients with HF exhibit a characteristic pattern of generalized cognitive dysfunction encompassing primarily memory (verbal and visual), working memory, attention, mental flexibility, and executive function. Most patients with HF fulfil criteria for MCI, defined as an objective deficit in cognitive function without signs of dementia and with limited impact on activities of daily living.^5^ MCI may be amnestic (memory-predominant) or non-amnestic (affecting other cognitive domains in isolation).^5^

Although the precise pathophysiological mechanisms underpinning the causal relationship between HF and cognitive decline remain incompletely understood, shared risk factors, including atrial fibrillation (AF), hypertension, obesity, and type 2 diabetes mellitus, and pathophysiological processes such as impaired cerebral perfusion, micro-embolic events, ischaemic syndromes, cerebral inflammation, blood–brain barrier disruption, and endothelial and neurovascular unit dysfunction are each implicated.^6^

Cognitive decline is an independent prognostic factor in patients with HF, significantly increasing the risk of hospitalization and mortality, diminishing quality of life, and posing major challenges for clinical management and the maintenance of appropriate therapeutic regimens. The coexistence of HF and CI leads to progressive loss of independence in activities of daily living, a marked reduction in health-seeking behaviour, and escalating healthcare expenditure.

A scientific statement from the Heart Failure Society of America highlighted that no specific interventions have yet been proven to improve cognition or delay the progression of CI in patients with HF,^4^ highlighting the complex interactions between the brain and the heart in cardiovascular disease.^7^ Comprehensive, multimodal care strategies must therefore incorporate systematic awareness of cognitive decline from the outset, with early recognition and thorough assessment serving as essential components for optimizing patient outcomes. While current HF guidelines acknowledge the relevance of CI in the context of frailty, communication challenges, and end-of-life decision-making, they lack specific recommendations for routine cognitive screening or structured diagnostic protocols in clinical practice.

This review is distinguished from previous overviews by its explicitly practical and clinical orientation. Rather than focusing predominantly on pathophysiology, it integrates the epidemiology and prognostic significance of CI with actionable guidance for everyday HF care, centred on a proposed cognitive screening workflow and an explicit link between cognition, self-care, and medication adherence. Its intended contribution is therefore to translate a rapidly expanding and mechanistically complex evidence base into a usable framework for clinicians involved in the routine care of patients with HF.

This article is a narrative rather than a systematic review. Relevant literature was identified through searches of PubMed/MEDLINE, Embase, and Scopus from database inception to May 2026, using combinations of the terms HF, CI, cognitive dysfunction, dementia, MCI, self-care, medication adherence, and cognitive screening. Priority was given to meta-analyses, systematic reviews, large prospective cohorts, randomized trials, and contemporary scientific statements and guidelines, supplemented by hand-searching of the reference lists of key articles. Studies were selected on the basis of relevance, methodological quality, and contribution to the clinical themes of this review rather than through a formal protocol-driven process, and the synthesis presented here should be interpreted accordingly.

For clarity, this review distinguishes between several related but conceptually distinct states. Mild cognitive impairment denotes an objective, usually chronic deficit in one or more cognitive domains that does not substantially compromise independence; dementia describes a more severe and progressive impairment accompanied by loss of functional autonomy; and delirium refers to an acute, often fluctuating disturbance of attention and awareness, characteristically precipitated by acute illness or HF decompensation and potentially reversible. These entities differ in their mechanisms, prognostic implications, and management, and acute cognitive fluctuations occurring during decompensation should not be equated with established chronic impairment. Unless otherwise specified, the term CI is used here to encompass the chronic spectrum from MCI to dementia.

### Epidemiology and clinical profile of cognitive impairment in heart failure

Cognitive impairment is a common and clinically significant comorbidity in HF. Epidemiological studies estimate that CI affects approximately 30%–50% of patients, although prevalence varies substantially depending on study populations and cognitive assessment methods employed.^8,9^ In some cohorts, the prevalence exceeds 50%, while clinically diagnosed dementia occurs in approximately 10%–20% of HF patients.^8^ Longitudinal analyses demonstrate that individuals with HF have a significantly increased risk of developing MCI and dementia relative to the general population.^8–10^

A recent meta-analysis including 12 112 older adults with HF (mean age 80.0 ± 4.9 years; 52.6% female) showed a prevalence of approximately 44%.^11^

Both major HF phenotypes, HF with reduced ejection fraction (HFrEF) and HF with preserved ejection fraction (HFpEF), are associated with cognitive decline, although through potentially distinct mechanisms. In HFrEF, reduced cardiac output may lead to chronic cerebral hypoperfusion, contributing to neuronal injury and progressive cognitive deterioration.^12^ In contrast, HFpEF is typically associated with older age and a high burden of cardiovascular comorbidities, including hypertension, diabetes mellitus, and AF, which may promote cerebral small-vessel disease and neurodegenerative processes.^10,12^

The prognostic relevance of CI in HFpEF has been demonstrated in the PARAGON-HF trial substudy of 2895 patients, in which 62.5% had normal cognitive function [Mini-Mental State Examination (MMSE; 28–30)], 27.4% showed borderline impairment (MMSE 24–27), and 10.1% were classified as cognitively impaired (MMSE <24).^13^ Even mild cognitive deficits were independently associated with increased risk of HF hospitalization and cardiovascular mortality, with adjusted hazard ratios of 1.27 (95% CI 1.06–1.53) for borderline impairment and 1.58 (95% CI 1.21–2.06) for MMSE <24.^13^

Neuroimaging studies further support a vascular contribution to cognitive decline in HFpEF. Subclinical cerebral infarctions have been identified in 29.3% of HFpEF patients without AF, compared with 17.3% in individuals without HFpEF, implicating covert cerebrovascular injury as a contributor to cognitive dysfunction.^14^ A clinical cohort study of 326 elderly HFpEF patients identified diabetes mellitus, prior stroke or transient ischaemic attack, carotid artery disease, elevated N-terminal pro-B-type natriuretic peptide (NT-proBNP), and reduced estimated glomerular filtration rate as independent predictors of CI, yielding a multivariable predictive model with good discrimination (area under the curve = 0.84).^15^

### Mechanisms linking heart failure and cognitive impairment

**Figure 1:**
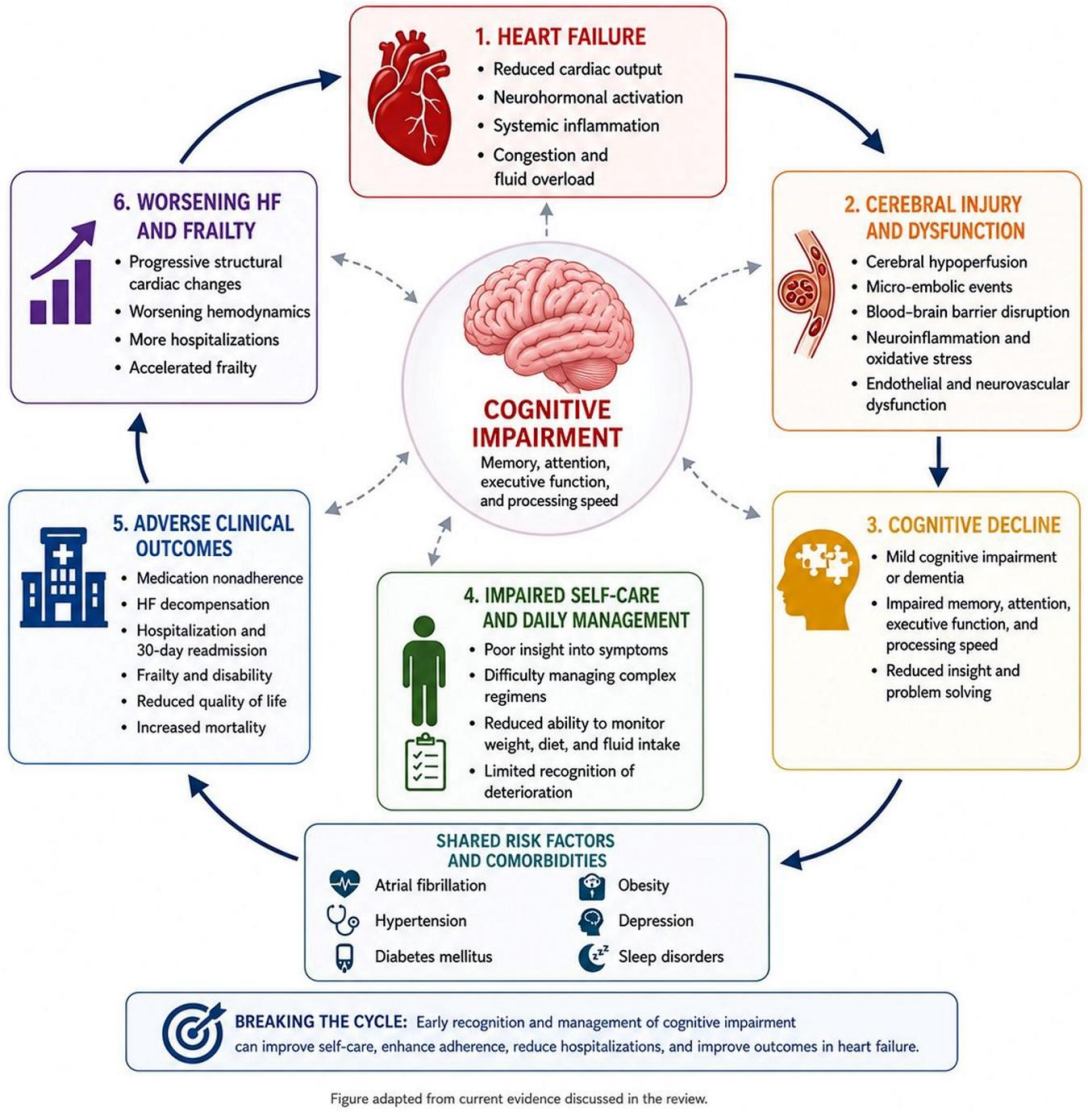
Mechanisms linking heart failure and cognitive impairment: a vicious cycle. CI, cognitive impairment; HF, heart failure

The pathophysiology of CI in HF is multifactorial and incompletely understood, reflecting the convergent effects of HF-related haemodynamic compromise, shared cardiovascular comorbidities, and non-cardiovascular pathological processes.^16^ Several interconnected mechanisms have been proposed, and their relative contributions likely vary according to HF phenotype, disease severity, and individual patient characteristics (*Figure 1*).

Cerebral hypoperfusion is the most extensively studied mechanism. In HFrEF, reduced cardiac output diminishes cerebral blood flow by an estimated 14%–30%, producing chronic cerebral ischaemia and progressive neuronal injury.^10^ Reduced systolic blood pressure and impaired cerebrovascular autoregulation further amplify this effect, often producing diffuse injury across multiple brain regions.^17^ Importantly, HF independently predicts both coronary and cerebral microvascular dysfunction, with impaired cerebral oxygenation reserve correlating significantly with lower cognitive scores, a relationship driven by global haemodynamic compromise rather than ejection fraction *per se*.^18,19^ The absence of a consistent universal association between cerebral blood flow and cognition across studies, however, suggests that hypoperfusion alone does not fully account for the CI observed in HF.^12^

Beyond haemodynamic mechanisms, systemic neuroinflammation is increasingly recognized as a critical contributor. Pro-inflammatory cytokines, including TNF-α, IL-6, and IL-1β, released from the failing myocardium gain access to the central nervous system, particularly when blood–brain barrier integrity is compromised, and activate neuroinflammatory cascades that accelerate neuronal injury.^20,21^ This process is compounded by oxidative stress: upregulated angiotensin II promotes reactive oxygen species production, further disrupting blood–brain barrier function and facilitating amyloid-β accumulation, a hallmark of neurodegenerative pathology.^20,22^

Neurohumoral activation exerts direct adverse effects on the brain. Chronic sympathetic overdrive and sustained activation of the renin–angiotensin–aldosterone system impair cerebrovascular autoregulation and may directly damage hippocampal neurones.^20,22^ Comorbidities frequently co-occurring with HF, including AF, hypertension, and atherosclerotic vascular disease, independently contribute to cognitive decline through cerebral small-vessel disease, large-artery occlusion, and cardioembolic events.^16^ Structural neuroimaging provides corroborating evidence: grey matter atrophy, white matter hyperintensities, medial temporal lobe volume loss, and subclinical cerebral infarctions have each been identified in HF patients and correlate with the degree of cognitive dysfunction.^10,12^

Collectively, these findings indicate that CI in HF arises from a complex interplay of haemodynamic compromise, neuroinflammation, oxidative stress, neurohumoral dysregulation, and cerebrovascular injury, rather than from any single pathological pathway. Although these mechanisms operate across the HF spectrum, their relative contributions are likely to be phenotype-dependent. Cerebral hypoperfusion secondary to reduced cardiac output is most directly relevant to HFrEF, whereas cerebral small-vessel disease and comorbidity-driven injury, reflecting the older age and higher burden of hypertension, diabetes, and AF characteristic of HFpEF, are thought to predominate in patients with preserved ejection fraction. Neuroinflammation, oxidative stress, and neurohumoral dysregulation appear to be shared across both phenotypes. This mechanistic complexity underscores the challenges in identifying targeted therapeutic strategies.

### Assessment of cognitive impairment in heart failure

The diagnostic assessment of CI in HF patients does not fundamentally differ from standard approaches used in non-cardiac populations, with both subjective (patient self-report and informant-based history) and objective (neuropsychological testing) measures remaining applicable across patient groups.

A cognitive history obtained from both the patient and a reliable informant (family member or caregiver), may not be sufficient to establish a formal diagnosis. Moreover, cognitive changes are frequently misattributed to normal ageing rather than underlying CI, particularly in elderly patients.

Nonetheless, such reports should be regarded as significant clinical red flags warranting comprehensive evaluation. Clinical suspicion should equally be raised by unexplained falls, medication errors, progressive dependence in instrumental activities of daily living, or the emergence of depressive symptoms or isolation. Confounding factors, including mood disorders, sleep disturbances, and delirium, must be carefully considered and, where clinically appropriate, addressed before cognitive test results are interpreted.

Several validated screening instruments are available for incorporation into routine clinical practice, selected according to available time, resources, and clinical context (*Table 1*). In the absence of consensus on the optimal screening tool for HF patients, instrument selection should be guided not only by pragmatic considerations but also by the differing diagnostic performance characteristics of available measures.

**Table 1:** Comparison of validated cognitive screening instruments for use in patients with heart failure

	MMSE	MoCA	Mini-Cog
Administration time (min)	8–10	10–15	3–5
Education bias	High	Moderate	Low
Training required	Yes	Yes	No
Number of items	11	16	2
Cognitive domains assessed	Orientation Attention/short-term memory Language Visuo-spatial	Orientation Attention/short-term memory Language Visuo-spatial Executive function	Visuo-spatial Short-term memory
Activities required	Drawing Counting Reading Writing	Drawing Counting	Drawing
Scoring range	0–30	0–30	0–5
Score interpretation	24–30: normal 18–23: mild impairment 10–17: moderate impairment <10: severe impairment	26–30: normal 18–25: mild cognitive impairment 10–17: moderate cognitive impairment <10: severe cognitive impairment	0–2: cognitive impairment likely 3–5: cognitive impairment less likely
Sensitivity for MCI	Low-Moderate	High	Moderate

The score ranges shown are indicative rather than absolute. Published cut-offs vary across validation studies and instrument versions, and both test performance and the interpretation of any given score are influenced by age, educational attainment, language, cultural background, sensory impairment, acute illness or delirium, and depression. A MoCA score below 26 is conventionally regarded as abnormal, with one point commonly added for individuals with 12 or fewer years of formal education. Screening results should, therefore, be interpreted in clinical context and confirmed, where appropriate, by formal neuropsychological assessment.

MMSE, Mini-Mental State Examination; MoCA, Montreal Cognitive Assessment.

Interpretation of cognitive screening results in HF is liable to confounding by several coexisting factors. Depression is prevalent in HF and is independently associated with deficits in attention, memory, and executive function that may compromise test performance irrespective of underlying neurological pathology.^23^ Delirium, particularly in the context of acute hospitalization or HF decompensation, can produce transient cognitive disturbances that superficially mimic or unmask pre-existing CI.^24^

Educational attainment and cultural background can significantly influence performance on standardized cognitive tests, potentially resulting in misclassification, particularly in individuals with lower educational levels or those from culturally diverse backgrounds.^25,26^

**Figure 2:**
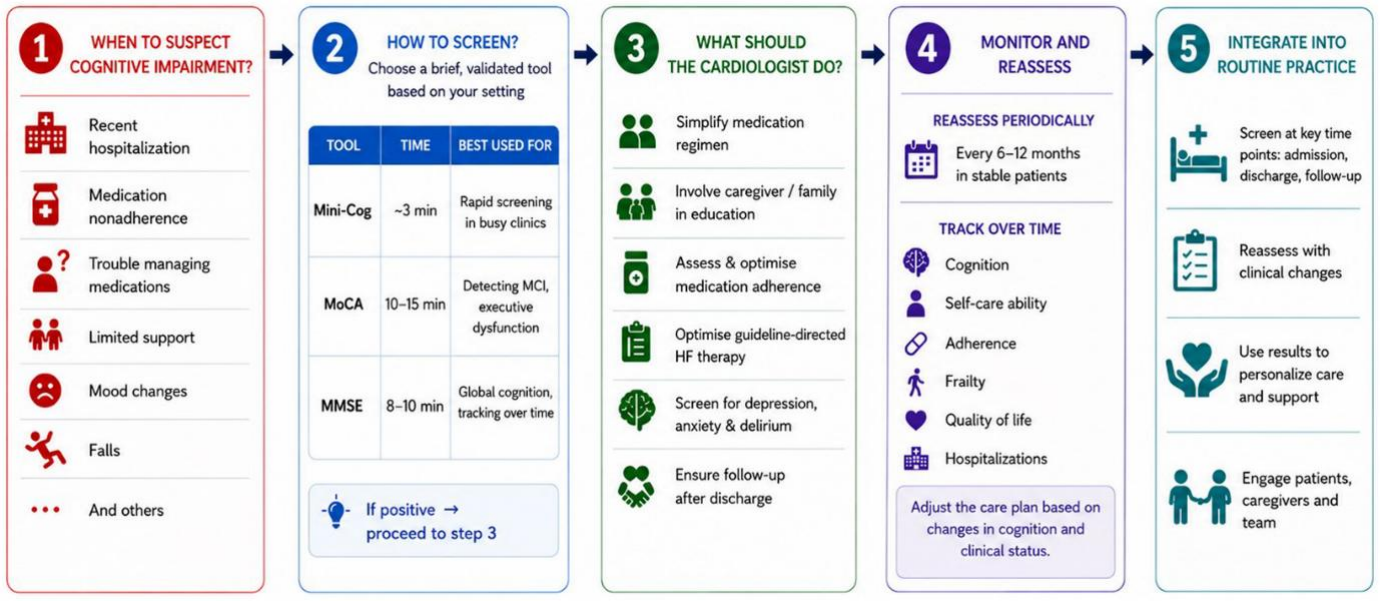
Practical cognitive screening in heart failure: a clinical workflow for cardiologists. CI, cognitive impairment; HF, heart failure; MCI, mild cognitive impairment; MoCA, Montreal Cognitive Assessment; MMSE, Mini-Mental State Examination

Practical recommendations for cognitive screening in HF include considering screening in all patients, with particular emphasis on those aged 65 years or older, or with a history of CI (*Figure 2*).^16^ Routine evaluations should be performed at the time of initial HF diagnosis and repeated periodically thereafter, with heightened vigilance following significant clinical events such as acute hospitalizations.^5^ Where screening identifies probable CI, referral for comprehensive neuropsychological evaluation is advisable to characterize the extent, nature, and pattern of deficits, and to guide individualized clinical management.^16,27^

### Cognitive impairment, self-care, and medication adherence

Patients with HF are at substantially increased risk of CI, with a recent meta-analysis estimating prevalence at approximately 41%–42% in this population.^8^ CI is particularly prevalent among older patients hospitalized for acute decompensated HF, the majority of whom demonstrate Montreal Cognitive Assessment (MoCA) scores consistent with cognitive deficits.^28^ Cognitive impairment frequently goes unrecognized in clinical practice and may substantially impair effective disease management, self-care, and adherence to therapeutic recommendations.^28^

Self-care can be conceptualized as a sustained process of maintaining health through health-promoting, preventive, and illness-management practices. Effective self-care is associated with improved quality of life, reduced rates of hospitalization, and lower mortality in HF patients.^29^ However, HF self-care demands considerable and sustained cognitive effort: patients must daily monitor symptoms (breathlessness, peripheral oedema), regulate fluid intake and physical activity, and maintain complex self-maintenance behaviours including strict medication adherence.^30^ These activities require intact capacities for cue recognition, clinical decision-making, illness comprehension, and self-management proficiency.^31^

Patients with CI experience particular difficulty understanding health information, sustaining attention on specific tasks, and processing complex clinical details sufficiently to make informed decisions regarding their health. In such contexts, caregivers assume a central role in supporting the patient and facilitating effective self-care. Caregiver engagement and education are therefore integral to the management of HF in the presence of CI.

### Moderators and mediators of the relationship between cognition and adherence

The relationship between CI and medication adherence in HF is modulated by a complex interplay of psychosocial, social, and structural factors.^32^ Depression and mood disturbance, prevalent comorbidities in HF, mediate the association between CI and adherence by impairing motivational capacity and executive planning, both of which are critical for maintaining complex pharmacological regimens.^33^ Social support and caregiver involvement moderate this relationship by providing external structure, reminders, and oversight, thereby attenuating the adverse impact of cognitive deficits on medication-taking behaviour.^34^ Health literacy and self-efficacy serve as further mediators: patients with greater understanding of their condition and higher confidence in self-management demonstrate superior adherence despite the presence of CI. Conversely, therapeutic complexity and polypharmacy substantially amplify adherence challenges for cognitively impaired patients, underscoring the clinical importance of regimen simplification and implementation of tailored educational or reminder-based interventions.^35^

### Clinical consequences of non-adherence

Medication non-adherence in HF, typically defined as administration of fewer than 80% of prescribed doses, is associated with significantly elevated rates of all-cause mortality and cardiovascular hospitalization. In one community-based cohort, non-adherent patients demonstrated more than twice the risk of combined death and hospitalization relative to adherent counterparts.^36^ Poor adherence results in inadequate neurohormonal blockade, progressive symptom deterioration, recurrent haemodynamic decompensation, and more frequent emergency healthcare utilization. Prospective analyses confirm that suboptimal adherence mediates the relationship between HF symptoms and reduced cardiac event-free survival, highlighting its central role in disease progression.^37^ At a systemic level, non-adherence substantially increases healthcare costs and indirect societal burdens attributable to lost productivity.^38^

These findings collectively underscore the need for routine cognitive and mood screening in HF clinical practice, together with multifaceted adherence-support strategies tailored to the individual patient’s cognitive profile. Evidence suggests that interventions based on frequent interpersonal contact are superior to automated reminder systems alone in improving pharmacotherapy adherence among cognitively impaired patients.^39^

### Cognitive impairment and prognosis in heart failure

Cognitive impairment is an independent predictor of mortality in HF, with a consistent and clinically significant association demonstrated across prospective cohort studies and meta-analyses. A meta-analysis of 12 studies encompassing 9556 patients demonstrated that CI was associated with an 88% increase in all-cause mortality risk.^40^ This relationship follows a severity gradient: severe CI confers more than double the mortality hazard compared with cognitively intact patients in adjusted analyses.^41^ The association is consistent across HF phenotypes; in HFpEF, even modest baseline CI independently predicts a graded increase in HF hospitalization and cardiovascular death, with cognitive decline during follow-up predicting mortality independently of baseline cognitive status.^13^ Notably, CI is associated with increased mortality risk even in younger and middle-aged HF patients, challenging the assumption that this prognostic association is confined to elderly populations.^42^ Sex may further modulate risk: CI predicts a 2.4-fold greater increase in cardiovascular mortality in women compared with men.^43^ These findings establish CI as a clinically meaningful prognostic variable warranting routine integration into HF risk stratification.^20^

Cognitive impairment is strongly and consistently associated with increased hospitalization and rehospitalization in HF. A systematic review and meta-analysis demonstrated that CI significantly increases the risk of 30-day rehospitalization, an effect that persists even when analyses are restricted to patients without overt dementia.^44^ Patients with HF and CI demonstrate a 30-day readmission rate of 26.8%, compared with 13.2% in cognitively intact patients.^45^ Even mild CI in the setting of acute HF is associated with significantly worse short-term outcomes relative to normal cognition, underscoring that the prognostic burden is not restricted to severe or formally diagnosed dementia.^46^ Despite this, CI is formally documented in fewer than 9% of medical records, meaning its contribution to readmission risk is systematically underestimated in routine clinical practice.^45^

Cognitive impairment poses significant challenges to safe care transitions following HF hospitalization, particularly following acute admissions where CI may first become clinically apparent.^42^ Structured post-discharge disease management programmes, encompassing early clinical review, medication reconciliation, and systematic telephone follow-up, reduce 30-day readmission rates and mortality in HF patients with CI, with evidence of particular benefit in those meeting criteria for MCI.^47^ Selected key studies spanning the domains of prevalence, prognosis, and management are summarized in *Table 2*.

**Table 2:** Summary of selected key studies of cognitive impairment in heart failure, by domain

Study (author(s), year)	Design/domain	HF phenotype	Cognitive tool	Sample size	Main finding
Cannon *et al.* 2017^4^	Systematic review and meta-analysis; prevalence	Mixed (HFrEF/HFpEF)	Various	Pooled cohorts	Pooled prevalence of CI ≈40%
Yap *et al.*, 2022^8^	Systematic review, meta-analysis and meta-regression; prevalence	Mixed	Various	Multiple cohorts	Prevalence of CI ≈41%–42%; increased dementia risk
Takagi *et al.*, 2026^11^	Global pooled meta-analysis; prevalence	Mixed (older adults)	Various	12 112	Prevalence ≈ 44% (mean age 80 years)
Shen *et al.*, 2024 (PARAGON-HF)^13^	Trial substudy; prognosis	HFpEF	MMSE	2895	Graded increase in HF hospitalization and CV death with lower MMSE
Cogswell *et al.*, 2017^14^	Cohort; neuroimaging	HFpEF	Brain MRI	Case–control	Subclinical cerebral infarction in 29.3% of HFpEF patients
Arnautu *et al.*, 2025^15^	Cross-sectional; predictors and risk score	HFpEF	MMSE-2	326	Clinical risk score for CI (AUC 0.84)
Zhang *et al.*, 2022^40^	Meta-analysis; mortality and readmission	Mixed	Various	9556	CI associated with 88% higher all-cause mortality
Kewcharoen *et al.*, 2019^44^	Systematic review and meta-analysis; readmission	Acute HF	Various	Multiple cohorts	CI increases 30-day rehospitalization, including without dementia
Agarwal *et al.*, 2016^45^	Prospective cohort; readmission	Mixed	Mini-Cog	241 encounters	30-day readmission 26.8% (CI) vs 13.2% (intact)
Miao *et al.*, 2024^42^	Prospective cohort; prognosis in younger patients	Acute HF	Mini-Cog	1958	CI in 19.6%; higher short-term adverse events
Dolansky *et al.*, 2016^35^	Prospective cohort; medication adherence	Mixed	Neuropsychological battery	309	Poorer cognition predicts lower objectively measured adherence
Huynh *et al.*, 2021^47^	Prospective cohort; management response	Mixed	Cognitive screening	Post-discharge cohort	CI modifies response to post-discharge management programmes

AUC, area under the curve; CI, cognitive impairment; CV, cardiovascular; HF, heart failure; HFpEF, heart failure with preserved ejection fraction; HFrEF, heart failure with reduced ejection fraction; MMSE, Mini-Mental State Examination; MRI, magnetic resonance imaging.

### Therapeutic implications

The therapeutic management of CI in HF presents a considerable clinical challenge. A scientific statement from the Heart Failure Society of America acknowledged that no specific pharmacological or non-pharmacological interventions have yet been demonstrated to improve cognition or reliably slow its progression in HF patients, underscoring the urgent need for rigorously designed, adequately powered clinical trials specifically addressing cognitive outcomes in this population.^16^ Accordingly, the strategies discussed below should be understood as biologically plausible and, in several instances, indirectly supported by surrogate or non-cognitive endpoints, rather than as cognition-specific, evidence-based recommendations; this distinction is made explicit throughout this section.

Optimized guideline-directed medical therapy (GDMT) for HF may confer indirect cognitive benefit by attenuating cerebral hypoperfusion and systemic neuroinflammation. Agents with neurohormonal effects, including angiotensin-converting enzyme inhibitors, angiotensin receptor-neprilysin inhibitors (ARNIs), beta-blockers, mineralocorticoid receptor antagonists, and sodium-glucose cotransporter-2 (SGLT2) inhibitors, may reduce the haemodynamic and inflammatory burden on the brain, although prospective evidence specifically for cognitive outcomes remains limited.^48^ The pleiotropic anti-inflammatory, anti-oxidative, and haemodynamic properties of SGLT2 inhibitors represent a particularly compelling target for future investigation of potential neuroprotection in HF.

Exercise-based cardiac rehabilitation has demonstrated potential cognitive benefits in HF. Aerobic training improves cerebral blood flow, attenuates systemic inflammation, and modestly improves performance across multiple cognitive domains.^21^ The HF-ACTION trial provided foundational evidence that structured exercise is safe and improves health status in chronic HF, though the trial was not designed to assess cognitive endpoints. Participation rates in cardiac rehabilitation programmes remain disproportionately low among cognitively impaired patients; dedicated, adequately powered trials with pre-specified cognitive endpoints are therefore required.

Simplification of pharmacological regimens represents a pragmatic and evidence-informed strategy for improving medication adherence in patients with CI. Approaches including once-daily fixed-dose combinations, compliance-aide packaging, dose administration aids, and pharmacist-led medication review have demonstrated efficacy in reducing medication errors among older adults with CI. Systematic medication reconciliation should be integral to every care transition in this population.

Multidisciplinary, patient-centred care models are essential to address the complex interaction between HF management, cognitive decline, and self-care capacity. Such models should incorporate specialist HF nurses, clinical pharmacists, neuropsychologists, social workers, and occupational therapists, with caregiver education and empowerment embedded as core components. Structured post-discharge follow-up programmes encompassing early clinical review, remote monitoring, and facilitated self-management are associated with reduced readmission rates in HF patients with CI and represent best current practice in the absence of disease-specific cognitive therapies.^47^

### Limitations and controversies

Despite accumulating evidence supporting the clinical significance of CI in HF, several methodological limitations and unresolved controversies merit careful consideration.

A fundamental confound arises from the frequent co-occurrence of depression in patients with HF. Elevated cortisol concentrations associated with depressive illness may independently impair cognitive performance, making it difficult to attribute observed cognitive deficits solely to HF-related pathophysiology.^49^ Furthermore, effective treatment of comorbid depression may partially ameliorate CI in HF, raising the possibility that a proportion of reported cognitive dysfunction reflects reversible, mood-related functional impairment rather than irreversible structural neurological injury.^49^

A second limitation concerns the generalizability of existing self-care measurement instruments in cognitively impaired HF populations. No self-care scale has been comprehensively validated specifically for patients with significant CI, and the interpretation of self-care data in this subgroup may therefore be systematically misleading.^50,51^

Commonly used cognitive screening instruments each carry important caveats in the HF setting. Although some studies have reported that the MMSE may perform comparably to, or in selected older HF populations better than, the MoCA for identifying CI,^52^ the relative diagnostic value of the two instruments depends on the population studied, the cognitive domain of interest, and the purpose of testing; both instruments are also susceptible to confounding by educational attainment, cultural background, and acute illness or delirium, factors particularly prevalent in hospitalized HF populations. The absence of a disease-specific, validated cognitive screening tool optimized for HF represents a significant unmet need.

Finally, marked heterogeneity across studies, in patient populations, HF phenotypes, cognitive assessment methods, CI definitions, and confounder adjustment strategies, limits the comparability of findings and the strength of conclusions drawn from pooled meta-analytic data. Prospective, longitudinal studies employing standardized assessment protocols and rigorous adjustment for key confounders are required to clarify the natural history and trajectory of cognitive decline in HF, and to identify modifiable determinants amenable to targeted intervention.

## Conclusions

Cognitive impairment is a prevalent, underdiagnosed condition that is independently and significantly associated with increased all-cause mortality, higher rates of hospitalization and 30-day readmission, impaired self-care, reduced medication adherence, and diminished quality of life.

Routine cognitive and mood screening using validated, time-efficient instruments, such as the Mini-Cog, MoCA, or MMSE, is a reasonable and increasingly advocated component of HF clinical assessment, particularly at the time of initial diagnosis and following acute decompensations; at present, however, this reflects expert opinion and pragmatic clinical consensus rather than a guideline-mandated requirement, as current HF guidelines do not specifically recommend routine cognitive screening. Clinicians must remain attentive to the confounding effects of depression, delirium, and educational background when interpreting screening results, and should maintain a low threshold for referral to specialist neuropsychological services when CI is suspected.

Although no pharmacological or non-pharmacological intervention has yet been demonstrated to specifically improve or preserve cognition in HF, several rational strategies merit adoption in current practice: optimization of GDMT to attenuate haemodynamic and neuroinflammatory cerebral burden; structured exercise rehabilitation; simplification of pharmacological regimens to reduce the cognitive demands of adherence; and multidisciplinary care models that embed caregiver education, systematic adherence support, and coordinated post-discharge follow-up. These approaches represent best current practice in the absence of disease-specific cognitive therapies.

Future research priorities should include prospective, longitudinal studies with standardized cognitive assessment protocols; rigorous evaluation of the cognitive effects of emerging HF therapies, including SGLT2 inhibitors and ARNIs; development and validation of HF-specific cognitive screening instruments; and randomized controlled trials of targeted cognitive and behavioural interventions. Integrating CI systematically into HF risk stratification and clinical decision-making represents a critical and currently unmet imperative for the comprehensive, patient-centred care of this complex and growing patient population.

Future research should evaluate whether systematic psychological assessment and targeted behavioural interventions can improve self-care, adherence to therapeutic recommendations and, ultimately, clinical outcomes in this vulnerable population.

## Declarations

### Data Availability

No data were generated or analyzed for this manuscript.
